# A New Syringe

**Published:** 1887-07

**Authors:** 


### A New Syringe.

A very neat and efficient syringe has been devised by Dr. J. A. Dunn of Chicago. It consists of a terminal point or portion similar in form to the ordinary hypodermic syringe ; instead of the ordinary cylinder, a small elastic rubber bulb is used. The point is made of platinum or gold, so that any acid that may be desired can be used. It is easily kept in order, and is very easily introduced into irregular canals or tracts. This is a very valuable instrument in the treatment of alveolar abscess, the roots of pulpless teeth, and pyorrhoea-alveolaris. This instrument should be in the hands of every dentist.

